# Potential Roles of Extracellular Vesicles as Biomarkers and a Novel Treatment Approach in Multiple Sclerosis

**DOI:** 10.3390/ijms22169011

**Published:** 2021-08-20

**Authors:** María Gutiérrez-Fernández, Fernando de la Cuesta, Antonio Tallón, Inmaculada Cuesta, Mireya Fernández-Fournier, Fernando Laso-García, Mari Carmen Gómez-de Frutos, Exuperio Díez-Tejedor, Laura Otero-Ortega

**Affiliations:** 1 Neurological Sciences and Cerebrovascular Research Laboratory, Department of Neurology, Neuroscience Area of IdiPAZ Health Research Institute, La Paz University Hospital, Universidad Autónoma de Madrid, 28046 Madrid, Spain; mariagutierrezfdez@hotmail.com (M.G.-F.); antonio.tallon@salud.madrid.org (A.T.); inmapuertas@hotmail.com (I.C.); fernandezfournier@hotmail.com (M.F.-F.); fernilaso9@hotmail.com (F.L.-G.); mcarmen.gomezf@gmail.com (M.C.G.-d.F.); 2 Department of Pharmacology and Therapeutics, School of Medicine, Universidad Autónoma de Madrid, 28046 Madrid, Spain; fernando.delacuesta@uam.es

**Keywords:** antigen delivery system, biomarkers, response to treatment, extracellular vesicles, multiple sclerosis, vaccine-like treatment

## Abstract

Extracellular vesicles (EVs) are a heterogeneous group of bilayer membrane-wrapped molecules that play an important role in cell-to-cell communication, participating in many physiological processes and in the pathogenesis of several diseases, including multiple sclerosis (MS). In recent years, many studies have focused on EVs, with promising results indicating their potential role as biomarkers in MS and helping us better understand the pathogenesis of the disease. Recent evidence suggests that there are novel subpopulations of EVs according to cell origin, with those derived from cells belonging to the nervous and immune systems providing information regarding inflammation, demyelination, axonal damage, astrocyte and microglia reaction, blood–brain barrier permeability, leukocyte transendothelial migration, and ultimately synaptic loss and neuronal death in MS. These biomarkers can also provide insight into disease activity and progression and can differentiate patients’ disease phenotype. This information can enable new pathways for therapeutic target discovery, and consequently the development of novel treatments. Recent evidence also suggests that current disease modifying treatments (DMTs) for MS modify the levels and content of circulating EVs. EVs might also serve as biomarkers to help monitor the response to DMTs, which could improve medical decisions concerning DMT initiation, choice, escalation, and withdrawal. Furthermore, EVs could act not only as biomarkers but also as treatment for brain repair and immunomodulation in MS. EVs are considered excellent delivery vehicles. Studies in progress show that EVs containing myelin antigens could play a pivotal role in inducing antigen-specific tolerance of autoreactive T cells as a novel strategy for the treatment as “EV-based vaccines” for MS. This review explores the breakthrough role of nervous and immune system cell-derived EVs as markers of pathological disease mechanisms and potential biomarkers of treatment response in MS. In addition, this review explores the novel role of EVs as vehicles for antigen delivery as a therapeutic vaccine to restore immune tolerance in MS autoimmunity.

## 1. Introduction

Multiple sclerosis (MS) is a chronic autoimmune and inflammatory disease affecting the central nervous system (CNS). MS is the main cause of nontraumatic neurologic disability in young adults, affecting approximately 2.5 million people worldwide [1]. The hallmark of this demyelinating disease is tissue injury arising from a complex interplay between the immune and nervous systems. Activated autoreactive T lymphocytes respond against one or more unidentified myelin protein, which induce inflammation characterised by innate and adaptive immune system cells causing demyelination, astrocyte reaction, blood–brain barrier (BBB) permeability, apoptosis of oligodendrocytes, and, ultimately, axonal loss and neuronal death in the CNS [2]. Innumerable questions remain regarding what triggers MS, and despite decades of effort, a cognate antigen that initiates the inflammatory process has not yet been identified. Interestingly, analyses of active lesions suggest that a single immune-effector mechanism dominates in each patient [3]. The heterogeneity of the disease highlights the importance of finding new biomarkers that characterise the disease in each patient, which will be crucial for improving MS management.

**Figure 1:**
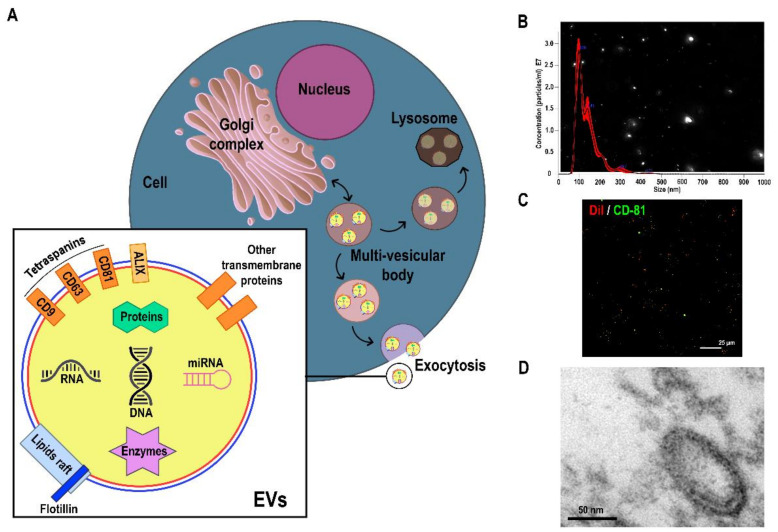
Structure and characterisation of EVs. (**A**) Scheme of formation, release, structure, and cargo of EVs. (**B**) Concentration and size of particles by nanoparticle tracking analysis in a sample of EVs. (**C**) Immunofluorescence of an EV sample with CD81 as a specific EV marker (green) and DiI (red) as a specific lipophilic tracker. (**D**) EV morphology acquired by transmission electron microscopy.

Extracellular vesicles (EVs) are small spherical vesicles bounded by a double-lipid bilayer between 30 and 150 nm which derive from the multivesicular body of the cell (Figure 1) [4]. They are released by all types of cells and participate in cell-to-cell communication [4]. In their membrane surface, they present specific proteins called tetraspanins [5] and lipid rafts with flotillins [6], in addition to other transmembrane proteins [7]. EVs carry different bioactive molecules inside [8] which are determined by the cell origin [9] and represent the key factor in their function of cell-to-cell communication [9,10]. The more common molecules are proteins, enzymes, DNA, RNA, and different types of RNAs (Figure 1) [9,11]. These molecules can be implicated in biological functions both in health and disease, including MS [12]. The first evidence of the involvement of EVs in MS dates back to 1989 [13]. Even though this discovery is 30 years old, the novel field of EVs holds enormous potential that has been only partially explored in the last ten years. The exponentially growing field of EVs is now inspiring the design of biomedical research studies on advancing their use as biomarkers. In this regard, EV levels and content can differentiate patients with MS from healthy controls (HCs) and correlate with disease activity and lesions found in magnetic resonance imaging (MRI) [14,15,16,17,18,19]. Moreover, EVs could play a theranostic role in critically ill patients [20] and may predict the worsening of MS [17]. Recent evidence suggests there are novel subpopulations of EVs according to cell origin, such as those derived from nervous and immune systems, which provide information on the pathology of MS. These EVs could play a role as biomarkers that provide insight into disease activity and progression and that can differentiate patients’ disease phenotype. This information could enable new pathways for therapeutic target discovery, and consequently the development of new treatments. In this regard, current disease modifying treatments (DMTs) for MS have been shown to modulate circulating EVs in patients with MS [21]. This evidence suggests that repeatedly assessing EVs could provide real-time information about DMT responses in MS. Consequently, EVs might also help identify the most appropriate DMT for each patient and monitor their response, which would improve decisions concerning treatment initiation, choice, escalation, and withdrawal.

Over the last few years, EVs have been considered to be not only biomarkers, but also potential treatments for MS. For example, EVs from mesenchymal stem cells (MSCs) have been shown to promote brain repair and immunomodulation in experimental animal models of MS [22,23,24]. MSC-derived EVs could mediate their beneficial effects by delivering important key molecules, such as DNA, mRNA, non-coding RNAs, proteins, and lipids to recipient cells. Thanks to the development of nano-based delivery system technologies, EVs can be modified, engineered, or designed to carry a variety of therapeutic agents inside cells, which makes them excellent drug delivery vehicles [25]. Recently, engineering has modified EVs by loading myelin antigens inside, making them antigen-presenting platforms that can restore the antigen-specific peripheral immune tolerance of autoreactive T cells. This approach could be considered an “EV-based vaccine” for restoring immune tolerance, which could be a real revolution in the treatment for MS.

Without a doubt, the discovery of EVs has shown us a new direction for research in this autoimmune, demyelinating, and neurodegenerative disease. This review summarises the breakthrough role of nervous and immune system cell-derived EVs as markers of pathological mechanisms driving the disease and potential biomarkers of treatment response. Moreover, this review explores the novel role of EVs as vehicles for myelin antigen delivery in the form of “EV-based vaccines” as a new treatment approach for MS.

## 2. Progress in Isolation and Characterisation of Extracellular Vesicles

### 2.1. Methods for EV Isolation

While aiming to isolate EVs from biological samples, we must consider the following issues: (1) isolating pure EVs, without contaminants, is extremely challenging; (2) the greater the purity, the lower the yield; and (3) complete separation of EV subtypes (i.e., microvesicles from exosomes) is not possible due to the overlap in size and density ranges, as well as in the expression of surface biomarkers [26]. For these reasons, we have to assume that our EV preparations are enriched in EVs rather than pure samples. In addition, when using size- or density-based methods to separate EV subtypes, we will call the fraction enriched in microvesicles large EVs, and the fraction enriched in exosomes small EVs [27], given that both will possibly contain vesicles of the other subtypes.

Despite these limitations, when choosing the right isolation technique for the desired downstream application, we can extract robust results from previous experiments, considering the great potential of EVs in biomarker discovery [28], cell-to-cell communication research [29], and therapy [30]. Before isolating EVs, contaminating cells, debris, and larger particles must be removed, typically by a series of centrifugations. The range of techniques available for isolating EVs is broad and we will review these in detail next.

#### 2.1.1. Differential Centrifugation

To date, differential centrifugation is the most commonly used methodology in MS due to its ease of implementation and high yield. However, it has significant caveats (Table 1). These limitations are particularly relevant when working with biofluids, especially with blood plasma/serum, where contaminants (mainly protein aggregates and lipoproteins) can exceed a desirable proportion of the whole [31]. A combination of centrifugation at 10,000–12,000× *g* and a series of ultracentrifugations at 100,000–120,000× *g* can be performed to separate large EVs from small EVs, respectively [27]. Should there be no need to separate these, total EVs can be directly isolated using the latter process. The mechanical forces affecting EVs during ultracentrifugation can damage EVs, which is particularly relevant when they are used for subsequent functional analyses [26].

#### 2.1.2. Density-Gradient Ultracentrifugation

Density gradient ultracentrifugation (DGU) is performed in a similar manner to the conventional approach; in this case, however, the sample is applied on top of a cushion of sucrose or iodixanol [32]. The application of a density gradient leads to a much higher purity than the differential ultracentrifugation, but it comes at the expense of a lower yield and a greater complexity of the procedure.

#### 2.1.3. Size-Exclusion Chromatography

Size-exclusion chromatography (SEC) is easy to implement in a lab, is scalable, and offers moderate purity and yield [33], which can fulfil the needs of most sample origins and downstream approaches. The EVs are isolated by size by crossing through a chromatographic column packed with fine, porous beads composed of dextran polymers. Its biggest advantage is ensuring the integrity of the isolated EVs, which is recommended when they will be used in further functional analyses [34]. Its main disadvantages are its limited sample capacity and that it results in a substantial sample dilution; thus, it is usually combined with ultrafiltration before and after the procedure [33].

#### 2.1.4. Bidimensional: DGU + SEC

Bidimensional isolation based on density and size substantially improves the purity of the EV fraction by removing most contaminants [35]. It is recommended for applications in which contaminants might drastically interfere or when the sample is very complex, as in the case of blood plasma/serum [36]. However, although it achieves very high purity, this is a time-consuming and difficult-to-implement technique that results in a low yield.

#### 2.1.5. Precipitation

Precipitation is the process of transforming dissolved EVs into an insoluble solid. This method is rapid, inexpensive, highly scalable, does not require specialised equipment, and is easy to implement [32]. Its main limitation is that it represents the lowest purity of all techniques reviewed here. Therefore, it should be used for applications not requiring much purity (i.e., therapy when a beneficial effect is demonstrated after such isolation and contaminants do not interfere).

#### 2.1.6. Immunoisolation

Isolation by immunoaffinity capture is an interesting technique that offers the possibility of isolating specific EV subtypes based on surface markers [37], unlike other techniques. It is rapid, does not require specialised equipment, and provides high purity, but is also low yield and high cost. This technique is used to isolate EVs by specifically addressing their cell origin.

#### 2.1.7. Other Techniques

There are additional methodologies that efficiently isolate/enrich EVs, such as microfluidic devices, ion exchange chromatography, or tangential flow filtration. Novel techniques under development are field-flow fractionation (FFF), alternating current electrophoretics, acoustics, microfiltration, deterministic lateral displacement, lipid affinity technologies, and hydrostatic filtration dialysis.

### 2.2. Characterisation of EVs

#### 2.2.1. Detection by Biomarkers

To achieve proper isolation/enrichment of EVs, it is mandatory to provide protein quantification of specific markers. The most commonly used markers are transmembrane proteins from the tetraspanin family, such as CD63, CD81, CD9, and flotillins (Figure 1); and components of the EV-biogenesis machinery, such as ALIX and TSG101 [38]. These are more often quantified by Western blot analyses, but high-resolution flow cytometry (hFC) is increasingly used to combine markers’ detection and size distribution measurements.

#### 2.2.2. Determination of Size and Concentration

Single particle analysis techniques are required to obtain size distributions and particle concentrations. The most commonly used and approved by the scientific community are nano tracking analysis (NTA) (Figure 1) and tunable resistive pulse sensing (tRPS) [39]. Although dynamic light scattering is another option, it has demonstrated lower resolution of multimodal particle suspensions [40]; thus, it is not ideal for EV preparations. Although hFC can offer size distribution together with marker detection, concentration measurements are less accurate compared with NTA and tRPS [39].

#### 2.2.3. Visualisation

The usual method is transmission electron microscopy (Figure 1), which permits visualisation of particles with the typical cup-shaped morphology as well as evaluating their average size and heterogeneity [40]. If willing to obtain in vivo images shedding or uptake of EVs, super-resolution confocal microscopy is a very useful tool [41]. Membrane labelling methods can offer an idea of the biodistribution of EVs upon injection into animals or in vitro administration, but to secure transfer of cargo, more reliable methodologies are required, such as the cre-loxP imaging system [42].

## 3. EExtracellular Vesicles as Biomarkers of Brain-Immune Alliance: Intercellular Communication

**Figure 2:**
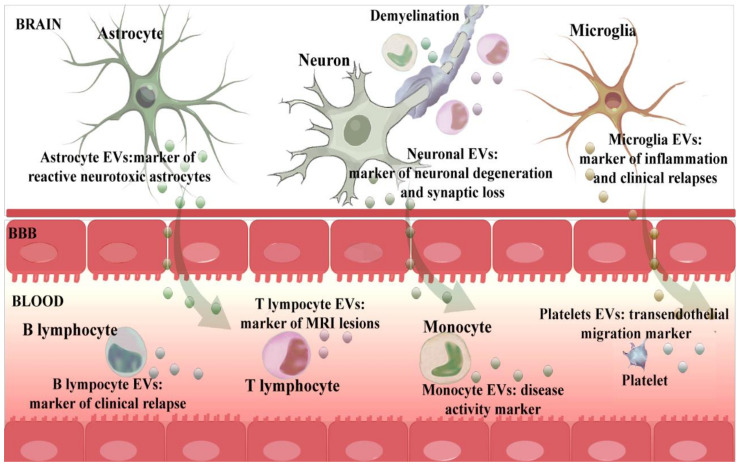
Circulating immune and brain-derived EVs as markers of the pathogenesis of MS. This figure shows EVs secreted by brain and immune cells that reach the bloodstream and play a role as circulating biomarkers of pathological processes of the disease.

The immune system and nervous system mutually interact in health and disease, forming a functional axis that involves EVs as a means of communication. In MS, brain cells secrete EVs that contain information regarding the pathological processes of the disease [25]. These nervous system-derived EVs can cross the BBB and reach the bloodstream [43,44]. Therefore, circulating EVs represent an accessible source of CNS biomarkers that act as a potential window into the pathological brain processes of MS. Moreover, immune system cells implicated in the inflammatory response also release EVs into the bloodstream that could reveal important information regarding the pathological reaction of the immune system that occurs in MS [25]. Taken together, circulating EVs are a mixture of vesicles originating from various types of cells that contain information about the pathological processes of the nervous and immune system. However, when analysing total circulating EVs, small changes occurring in EVs from cells involved in the pathogenesis of MS could be masked by dilution. Thus, a comprehensive study specifically addressing the cell origin of EVs and an in-depth study of those originating from the nervous and immune systems could provide more detailed information about the processes taking place in MS (Figure 2, Table 2). Due to a very small representation in the total circulating EV population, the analysis of nervous system-derived EVs in peripheral biofluids constitutes a formidable analytical challenge. It requires enrichment techniques such as the aforementioned immunoisolation, which might lead to peripherally detectable CNS biomarkers in MS [44].

Hereafter, we will summarise the studies that have been published thus far on nervous system and immune system-derived EVs in MS and their most important roles in the pathogenesis of the disease.

### 3.1. Neuronal-Derived EVs

The L1 cell adhesion molecule (L1CAM) had been identified as a promising marker for neuronal origin on blood circulating EVs in 2017 [44]. Some authors have used this marker to isolate neuronal-derived EVs from the blood in various pathologies, but this technique was not employed until 2021 to analyse neuronal-derived EVs in MS. The levels of L1CAM neuronal-derived EVs in patients with MS did not differ from those found in HCs. However, the neuronal-derived EVs’ average diameter was slightly greater in the MS group. In addition, synaptic markers in neuronal-derived EVs were markedly lower in patients with MS compared with HCs. These findings reveal that neuronal-derived EVs act as markers of the degree of neuronal degeneration and synaptic loss for each individual patient with MS [45].

### 3.2. Astrocyte-Derived EVs

Astrocyte-derived EVs from blood samples have also been isolated and were studied in patients with MS in 2021. Glutamate transporter primarily expressed by astrocytes (GLAST) has been used as a marker to isolate and analyse astrocyte-derived EVs from blood in MS patient samples. The concentration of GLAST-containing EVs was higher in HCs than in patients with MS, but their average diameter did not differ between groups. Multiple complement cascade components were markedly elevated inside astrocyte-derived EVs in the MS group compared with controls, including early (C1q, C3, C3b/iC3b) as well as late (C5, C5a) components and inhibitory factors (Factor H). This result was interpreted as a reflection of the increased abundance of reactive neurotoxic astrocytes in the disease. Consequently, the content of astrocyte-derived EVs could be considered a marker of neurotoxicity of astrocytes in the brain during MS [45]. These differences in complement content were not noted when comparing total circulating EVs, which highlights the importance of studying vesicles originating from different subpopulations, as these findings can be diluted if only total circulating EVs are analysed.

### 3.3. Microglia-Derived EVs

Circulating CD11b/c microglial-derived EVs were successfully isolated in an experimental autoimmune encephalomyelitis (EAE) animal model in 2012. The authors found that the levels of the microglia-derived EVs increased upon inflammation in the cerebrospinal fluid (CSF) and were closely associated with the course of the disease, peaking at onset and during clinical relapses, and decreasing in the chronic phase of the disease [46]. However, in patients with MS, an additional marker has been used to isolate microglial EVs in blood, IB4. In 2018, EVs were successfully isolated from microglia in the plasma of patients with MS using the IB4 marker, demonstrating that their levels increased during a stable phase compared with patients in the relapse phase [47].

### 3.4. Oligodendrocyte-Derived EVs

Oligodendrocytes also secrete EVs that can be detected in peripheral blood using oligodendrocyte-myelin glycoprotein markers [48]. However, despite the importance of the role of oligodendrocytes in MS, to our knowledge there are no studies that have isolated these vesicles from the blood of MS patients.

### 3.5. Endothelial-Cell Derived EVs

To gain insight into the role of EVs in MS, some authors have isolated endothelial-derived EVs from plasma and studied their role as biomarkers and their function on BBB permeability. The levels of CD31 endothelial-derived EVs could differentiate patients with MS from HCs and between MS types [49], and the levels depending on disease progression [50]. In particular, these researchers showed that high levels of endothelial CD31-positive EVs were present in the blood of patients with MS during disease exacerbation, whereas a reduction in the level of these EVs was observed during remission [49]. Moreover, these levels were found to correlate with MRI lesions in patients with MS [48]. While MRI remains a powerful imaging tool, endothelial-derived EV analysis could help refine the interpretation by providing additional information on MS [49]. Recent studies have demonstrated that brain endothelial cell-derived EVs provide a bridge protein (claudin-5) between leukocytes and endothelial cells that induces leukocyte transendothelial migration [51] and also facilitates transendothelial migration of monocytes to the brain parenchyma [52,53].

### 3.6. Monocyte-Derived EVs

Recent studies suggest that the innate immune system plays an important role in the initiation and progression of MS by influencing the effector function of T and B cells. Monocytes retained outside the brain do not contribute to CNS injury, but can interact and communicate via shedding vesicles with other cells, such as activated lymphocytes or natural killer cells [54]. EVs are important means through which myeloid cells exert their functions, and therefore they have been isolated from blood and studied by several authors [54]. Peripheral blood monocytes from patients with MS released higher levels of EVs than in HCs [54,55,56], and different amounts of EVs were found depending on the type of MS [21]. Patients with relapsing remitting MS presented significantly higher CD14 monocyte-derived EV levels than patients in the secondary progressive MS group [21]. Among relapsing remitting patients with MS, higher levels of EVs from monocytes were correlated with disease activity [57]. These findings indicate that monocyte-derived EVs could play a role in MS and might be a biomarker of disease type and disease activity.

### 3.7. T Lymphocyte-Derived EVs

The most important players in the pathogenesis of MS are anti-myelin CD4+ T lymphocytes [58]. The specific marker CCR5 has been used to isolate Th1-derived EVs and CCR3 to isolate EVs from Th2 cells in patients with MS. Patients with MS showed an increase in the levels of CCR5 Th1-derived EVs and CCR3 Th2-derived EVs in the presence of gadolinium-enhancing lesions in the brain and spinal cord [47]. These findings indicate that Th1 and Th2-derived EVs could act as markers of disease activity in MS. In addition, Tregs are also key players in MS. They release EVs, which contain a considerable amount of protein and RNA that regulate the proliferation or survival of T lymphocytes. However, the inhibitory effect of Treg-derived EVs was impaired in patients with MS, which contributes to the pathogenesis of MS. However, the cause of the EVs’ defect in patients with MS is unclear. Manipulation of patients’ Treg-derived EVs to restore their suppressive activity is a potential therapeutic approach [59].

### 3.8. B Lymphocyte-Derived EVs

B cell-derived EVs have also been associated with the pathogenesis of MS. Even though CD19+ B cell-derived EV levels did not differ between patients with MS and HCs, increased levels of these EVs were found in patients during the course of a clinical relapse compared with during remission [47]. Further investigation of the function of these EVs might provide information on the precise role of B-lymphocytes in MS pathogenesis.

### 3.9. Platelet-Derived EVs

Recently, evidence has emerged suggesting non-haemostatic roles for platelets, including inflammatory and immune responses. An association between platelets and MS was first indicated by the increased adhesion of platelets to endothelial cells in these patients [60]. During this process, platelets release EVs that interact with the underlying endothelium and contribute to the endothelial abnormalities involved in the pathophysiology of MS [61]. Sáenz-Cuesta et al. showed an increase in levels of CD41+ platelet-derived EVs in patients with MS compared with HCs [21], and these EVs were increased in all clinical forms of MS [62]. Platelet-derived EVs transport a variety of bioactive agents, including platelet activating factor, amyloid precursor protein, and complement factors, all of which could contribute to the disease process [61]. Moreover, platelet-derived EVs from patients with MS induced a strong disruption of endothelial barriers. These EVs should be considered not only as markers of early stages of MS, but also as pathological factors with the potential to increase endothelial permeability and leukocyte infiltration [62].

**Table 2:** Immune and brain-derived EV markers and their roles in MS.

**EV Marker**	Cell Origin	Roles in MS
L1CAM	Neuron	Marker of neuronal degeneration and synaptic loss degree for each individual patient [45].
GLAST	Astrocyte	Marker of the toxicity of astrocytes in each patient [45].
CD11b/c	Microglia	Marker of inflammation, disease course and relapse [46].
IB4	Microglia	Differentiating patients in the stable phase from the relapsing phase [47].
CD31	Endothelial cell	Differentiating MS patients from HC and between MS types [49]. Marker of disease exacerbation [41], remission [50] and transendothelial migration of leukocytes [51,52,53].
CD14	Monocyte	Differentiating MS patients from HC [54,55,56] and types of MS [21]. Marker of disease activity in relapsing remitting patients [57].
CCR5 CCR3	Th1 and Th2 lymphocyte	Markers of lesions found in MRI [47].
CD19	B lymphocyte	Differentiating patients with clinical relapse from those with remission [47].
CD41	Platelet	Inducing a strong disruption of endothelial barriers increasing endothelial permeability and leukocyte infiltration [62].

## 4. Extracellular Vesicles as Biomarkers of Disease Modifying Treatments’ Response/Failure

The past two decades are known as the treatment era in MS, as a variety of DMTs have been developed for MS based on modulation of the immune system. All of these DMTs aim to reduce the number of clinical relapses and the appearance of new lesions [63]. Given that the natural disease course of MS is unpredictable, the benefit of treatment for each patient is unknown and treatment decisions are complicated. Some patients treated with one DMT can continue to have relapses, inflammatory activity, and/or show irreversible neurological disability for which a therapy switch needs to be considered [64]. Due to this failing response to treatments, patient quality of life becomes reduced, increasing disability and healthcare costs. Currently, we do not understand why this happens or when it might happen. Thus, a biomarker that predicts the response to treatment and treatment failure could help with treatment decisions and would have an enormous positive impact on patients with MS. In this sense, the development of molecular diagnostic platforms is contributing to theranostic applications of EVs [20]. In this regard, circulating EVs have been shown to be modulated by ongoing DMTs, which might represent a new role for EVs as predictor biomarkers for treatment response [21,56]. In the next section, we review the studies that show how current DMTs affect circulating EV production and modification and the breakthrough role of these EVs as potential biomarkers of treatment response and adverse events:

### 4.1. EVs as Biomarkers of DMT Response

Interferon-beta (INF-β) was the first treatment approved for patients with MS. The mechanism of action of INF-β has not yet been completely identified, but it might promote suppression of T cell activity and prevent leukocyte transendothelial migration to the CNS [65]. The effect of INF-β on EVs was first explored in 2005. The authors showed that INF-β inhibited endothelial cell-derived EV production both in vitro and in vivo. Endothelial cell-derived EVs have been shown to enhance monocyte transendothelial migration to the CNS. Therefore, the authors conclude that INF-β blocks leukocyte entry into the CNS, at least in part, by inhibiting endothelial cell-derived EV production [52]. Along these lines, other studies have shown a reduction in the levels of endothelial cell-derived EVs after treatment with INF-β; unfortunately, these EV levels did not correlate with MRI findings at 12 weeks after treatment [53]. These results suggest that endothelial cell-derived EVs cannot act as biomarkers of either treatment response or disease activity in patients with MS. However, a subgroup of endothelial cell-derived EVs, in particular CD54+ endothelial cell-derived EVs, have been shown to correlate with MRI findings at 12 months [66]. Therefore, analysing CD54+ endothelial cell-derived EVs could provide information about response/failure to INF-β in patients with MS. These specific EVs provide information on whether INF-β is correctly preventing cell migration to the CNS, which is correlated with the lesions found on MRI. In addition, INF-β also reduced monocyte-derived EV production. This reduction started six months after treatment initiation and decreased further after twelve months [55]. This result shows that INF-β not only modifies endothelial cell-derived EVs but also those derived from monocytes. However, these results are controversial, given that other studies have reported an increase in the levels of plasma EVs derived from monocytes in INF-β-treated patients with MS. In addition to this result, an increase in the levels of plasma leukocytes and platelet-derived EVs were found in INF-β-treated patients with MS compared with non-treated patients [21]. Moreover, Dalla Costa reported that INF-β did not influence the monocyte-derived EV levels in patients with MS at three and twelve months after treatment initiation [54]. These controversial results suggest that an extensive study of the effects of INF-β on monocyte-derived EVs is needed to demonstrate whether these EVs could be used as biomarkers of response to INF-β. Given that the results of EV levels are controversial, further research analysing the profile of the EV cargo will be important. Differences in the miRNA profile were found in plasma-derived EVs of INF-β treated patients compared with naive patients. The microRNA found in INF-β-treated patients regulates T-cell activation and is involved in oligodendrocyte differentiation and in the demyelination and remyelination process [67].

Another DMT currently in use is natalizumab. Natalizumab is a recombinant humanised monoclonal antibody that prevents transendothelial cell migration to the CNS [68]. Natalizumab-treated patients with MS showed higher levels of plasma EVs derived from monocytes [54], leukocytes, and platelets compared with non-treated patients [21]. These higher levels of leukocyte-derived EVs in natalizumab-treated patients could be due to a blockage of leukocyte entry into the CNS, resulting in an increased number of leukocytes in the blood compartment and, in turn, their EVs [21].

Another approved DMT, fingolimod, induces downregulation of the sphingosine 1 phosphate receptors present in lymphocytes, reducing their egress from lymphoid tissues into the circulation. In that way, the drug reduces lymphocyte infiltration into the CNS. Acid sphingomyelinase (aSMase) is inhibited by fingolimod [69], and this enzyme controls EV production. Thus, an EAE animal model has shown that fingolimod can inhibit EV shedding, reducing the levels of myeloid cell-derived EVs in the CSF of EAE-treated mice. These EV levels correlated with neurological scores during fingolimod treatment [46]. Hence, a novel role for myeloid cell-derived EVs could be postulated, namely as a biomarker of response to fingolimod in EAE mice. Moreover, in patients with MS, fingolimod showed a reduction in monocyte-derived EVs after three and six months [54] as well as after twelve months of treatment [56]. However, when analysing total EVs from serum, fingolimod induced a two-fold increase in EV levels in serum of patients with MS and also induced a change in their microRNA cargo at five hours after treatment initiation. Thus, 26 microRNAs were overexpressed and nine under expressed at five hours after fingolimod administration. EVs obtained prior to fingolimod treatment showed increased immune regulatory activity compared with EVs obtained five hours post-treatment [21]. This outcome suggests that EVs could play a role in the mechanism of action of fingolimod from the initial hours and paves the way to explore a potential use for EVs in early treatment monitoring [21]. It has been identified that miRNAs specifically are altered in fingolimod responders compared with non-responders, and their impact on a variety of pivotal regulatory pathways has been predicted. This work suggests that EV miRNA profiles have the potential to be utilised in MS clinical practice as biomarkers of treatment response that can support personalised therapeutic decisions [70].

On the other hand, teriflunomide reduces the proliferation of B and T cells by inhibiting dihydroorotate dehydrogenase. Teriflunomide progressively reduces monocyte-derived EV production from the second month after drug administration, more significantly after six months, and even more at twelve months after treatment initiation. Therefore, the longer the patients were treated, the greater the decrease [55]. These results suggest that EVs could play a role in the mechanism of action of teriflunomide on monocytes after two months of administration and thereafter.

Finally, glatiramer acetate is an immunomodulatory agent that mimics myelin-basic protein with an artificial chemical structure [71]. Glatiramer acetate did not influence the monocyte-derived EV levels in patients with MS after three and twelve months of treatment [54].

### 4.2. EVs as Biomarkers of Adverse Events after DMTs

Natalizumab, dimethyl fumarate, fingolimod, alemtuzumab, and ocrelizumab have all been associated with cases of progressive multifocal leukoencephalopathy (PML) caused by JC polyomavirus (JCPyV). The measurement of plasma indexes of anti-JCPyV antibodies helps stratify patients treated with natalizumab who are at risk of PML [72], and this index is used as a clinical indicator that natalizumab needs to be changed or withdrawn. JCPyV infects the endothelial cells of the choroid plexus by attachment through specific receptors and infiltrates the brain parenchyma, infecting oligodendrocytes and astrocytes. However, the critical attachment receptor for the virus is paradoxically not expressed on oligodendrocytes or astrocytes in the human brain. JCPyV-infected choroid plexus epithelial cells produce EVs that contain JCPyV and readily transmit the infection to human glial cells [73]. Thus, JCPyV associates with EVs to infect target cells independently of virus receptors. This alternative mechanism of infection likely plays a critical role in the dissemination and spread of JCPyV both to and within the CNS [73]. Along these lines, an analysis of choroid plexus epithelial cell-derived EVs could provide real-time information about JCPyV infection. Choroid plexus epithelial cell-derived EV analysis could be an alternative strategy to the current indirect measurement of the risk of infection by anti-JCPyV antibodies. Therefore, EVs could act as biomarkers for discontinuation of the current DMT, targeting the real risk of JCPyV infection. Given that JCPyV takes over EV biogenesis pathways in infected cells, there has been evolving interest in trying to understand how EV cargo is being altered during JCPyV infections and how its transfer to surrounding uninfected cells could affect viral pathogenesis. The viral proteins found in EVs released from infected cells represent potential therapeutic targets [74]; therefore, EVs from choroid plexus epithelial cells could act not only as a biomarker of JCPyV infection, but also as a therapeutic target to treat the infection in the CNS [75].

All these findings suggest that EVs could be a real revolution in the prognostic field, given that they are easy-to-obtain biomarkers that can differentiate treatment response/failure and predict adverse events and disease progression. Without a doubt, the novel and exponential growing field of EVs is now inspiring the design of multiple biomedical research studies to advance their use as biomarkers of treatment response of this autoimmune, demyelinating, and neurodegenerative disease.

## 5. Role of Extracellular Vesicles as Treatment for Brain Repair and Immunomodulation in Multiple Sclerosis

Stem cell therapy is emerging as a novel and successful treatment for MS, given its potential for CNS repair and immunomodulatory properties. In particular, we have focused on MSCs that are adult stem cells with self-renewal ability located in bone marrow, adipose tissue, umbilical cord, and dental tissues that are easy to obtain and lack ethical concerns. They appear to be the most adequate sources of stem cells for the treatment of MS. The therapeutic potential of MSCs is associated with their antiapoptotic and anti-inflammatory properties as well as their potential for brain repair. Indeed, MSCs are able to secrete key molecules, such as growth and trophic factors, cytokines, microRNAs and proteins encapsulated in EVs [76]. These EVs might act as the active ingredients of MSCs. Evidence has demonstrated that the therapeutic potential of EVs is also similar to that of parental MSCs, which have unique advantages in terms of biodistribution (crossing the BBB and reaching the brain) and safety (avoidance of tumour formation and vascular occlusion); they can be stored in hospitals, and they avoid phagocytosis by macrophages and can circulate for extended periods of time within the body. The therapeutic potential of MSC-derived EVs has been evaluated in several experimental animal models of MS. The intravenous administration of EVs derived from MSCs has demonstrated improved motor deficits, reduced brain atrophy, increased cell proliferation in the subventricular zone, decreased inflammatory infiltration, and reduced inflammation in a Theiler’s murine encephalomyelitis virus (TMEV)-induced demyelinating disease used as a model of progressive MS [23]. Moreover, the effects of EVs derived from MSCs were also tested in an EAE model. The treatment decreased clinical severity scores [77] and reduced myelin oligodendrocyte glycoprotein (MOG)-induced proliferation of splenocytes, inflammatory infiltrates, and demyelination areas [78]. Intravenous injection of MSC-derived EVs also reduced neuroinflammation through regulation of microglia polarisation [79] and upregulation of the number of Tregs in the EAE spinal cords [80]. Moreover, other routes of administration seem to be valid and efficacious. Thus, intranasal administration of small MSC-derived EVs also showed a significant decrease in the clinical scores associated with an increase in immunomodulatory responses in EAE mice [81]. Furthermore, manipulation through bioengineering of the EV properties or cargo might lead to an enhancement of therapeutic efficiency. Thus, conjugation of the amine groups on the EV surface with the carboxylic acid-functionalised LJM-3064 aptamer (which has affinity for myelin) induced an enhancement of remyelination and a reduction in disease severity [82]. Moreover, MiR-219a-5p-enriched MSC-derived EVs induced oligodendrocyte progenitor cell differentiation and improved EAE animal model clinical evolution [83]. Lastly, interferon-treated MSCs release various miRNAs that can control microglia activation [84]. The results of these studies show the great potential of MSC-derived EVs in the treatment of MS.

## 6. Role of Extracellular Vesicles as Preventative Treatment for Multiple Sclerosis

As mentioned earlier, some viruses, including JCPyV, can hijack EV biogenesis systems for their dissemination, while EVs from infected cells can transfer viral proteins to uninfected cells. Due to their involvement in viral infections and the ability to deliver viral antigens to other cells, EVs have also been studied as potential therapeutic agents in the form of “EV-based vaccines” [74]. In the next section, we review the state-of-the-art EV-based vaccines for MS as a therapeutic approach to restoring immune tolerance in CNS autoimmunity.

In autoimmune diseases, the immune system is derailed and generates immunity against itself. Although the specific antigen has not been identified, myelin-derived antigens such as MOG, myelin basic protein (MBP), and proteolipid protein (PLP) have been proposed as key antigens promoting immune reaction [85]. A restoration of antigen-specific peripheral immune tolerance of autoreactive T cells could be an effective strategy for the treatment of MS. In 2021, Flemming developed a “vaccine-like treatment” for antigen-specific tolerisation of autoreactive T cells in a mouse model of MS [86].

The use of novel vehicles for vaccine delivery is now under investigation. Along these lines, lipid bilayer EVs could serve as prospective vaccine candidates, given that they are able to transfer their specific cargo, including antigens, to recipient cells. Hence, EVs facilitating the antigen delivery to monocytes, dendritic cells, macrophages, and microglia, which internalise antigen-presenting EVs, trigger antigen capture and induce immune tolerance [87]. This EV-based vaccine could act as an MS therapy based on restoration of antigen-specific peripheral immune tolerance. In particular, the antigen presentation to T cells by EVs is mediated through various processes, either through the dendritic cells or directly in T-cells. In the first model, EVs are efficiently internalised by the dendritic cells due to the presence of specific adhesion proteins on the EV surface. The dendritic cells can either present the entire EVs antigenic peptide–major histocompatibility complex (MHC) to the T cells (cross-dressing pattern), or EV antigens on their own MHC class I and II molecules (cross-presentation pattern). In the second model, due to the presence of MHC class I and II molecules on the EV surface, EVs present antigens directly to the CD4+ or CD8+ T-cells in the absence of dendritic cells [74].

The prerequisite for antigen-specific EV-based vaccines is knowledge of the relevant self-antigen targeted by the autoimmune response. However, the relevant self-myelin antigen in MS remains speculative, with the possibility that these antigens differ among patients and over time in the same patient. It has been shown that PLP is mainly present in small EVs, which are enriched in exosomes, whereas MBP and MOG are mainly present in large EVs, specifically the fraction enriched in microvesicles [88]. Given that EVs include exosomes and microvesicles, Casella et al. used total EVs from oligodendrocytes (which naturally contain most of the relevant myelin antigens) as an EV-based vaccine in various experimental animal models of MS. The intravenous injection of oligodendrocyte-derived EVs suppressed the clinical disease prophylactically and therapeutically in chronic and relapsing-remitting EAE models. This EV-based vaccine was safe and restored immune tolerance by inducing immunosuppressive monocytes and apoptosis of autoreactive CD4+ T cells. These findings introduce an approach to suppressing CNS autoimmunity in a myelin antigen-specific manner, without the need to identify the target antigens [88].

In addition to this strategy, immune-system derived-EVs have great potential as a novel EV-based vaccine to induce myelin antigen-specific tolerance. EVs released by antigen-presenting cells express MHC-I, MHC-II, and T cell costimulatory molecules that act as antigen presentation platforms [89]. These antigen-presenting cell-derived EVs could also be used as a vector to present myelin antigens to autoreactive T cells in the absence of costimulatory molecules, as a strategy for restoring immune tolerance in a myelin antigen-specific manner in MS [90]. EVs from the immune system are not naturally loaded with myelin antigens. Therefore, the artificial loading of the antigen of interest into the EVs is a requirement for the production of these therapeutic EV-based vaccines. This approach would involve EV engineering, and two main strategies are possible: (1) post-isolation EV engineering, in which EVs are directly modified after isolation from cells by electroporation, extrusion, sonication, incubation, freeze–thawing, bio-conjugation, click chemistry, or cloaking; and (2) parental cell-based EV engineering, in which donor cells are modified to obtain the EVs with the antigens of interest loaded into the EV lumen or displayed on the EV surface [91]. In addition to the parental cell-based EV engineering methods and devices developed for loading antigens of interest into EVs, similar methods have been developed for RNA loading. Recently, a novel parental cell-based strategy was established for loading mRNAs, called the EXOtic device [92]. RNAs can also be loaded into EVs, by fusing the above-mentioned proteins with RNA-binding proteins. The mRNA contained in EVs is transferred to the target cell and translated into the antigen of interest; in this way, these EVs act as antigen-presenting EVs. Engineering is designing more efficient vaccine EVs by increasing their stability, bioactivity, presentation to acceptor cells, and their capacity for on-target binding at both cell-type-specific and tissue-specific levels [74].

Taken together, EV-based vaccines overcome some limitations of conventional vaccines and introduce novel unique characteristics useful in vaccine design, including greater biosafety and efficiency as antigen-presenting systems and as adjuvants (Table 3). The characteristics are the following:

(1) Current DMTs for MS target the immune system in an antigen-nonspecific manner, and potentially induce serious adverse effects because of systemic immune suppression. Antigen-presenting EVs would only suppress harmful immune responses while leaving the rest of the immune system intact, thus reducing potential adverse effects [88].
(2) EVs present a low basal immunogenic profile. EVs mirror the immune antigenic signature of the producer cell. Along these lines, EVs derived from MSCs present a membrane surface with a low immunogenicity shape, with the absence or very low presence of immunogenicity markers, such as HLA-DR and HLA-ABC antigens. The low immunogenicity of the MSC-derived EVs guarantees the absence of immune system reactions when they are intravenously administered. This is a translatable clinical feature that makes MSC-derived EVs a better candidate for antigen-presenting biological nanoplatforms for patients with MS [92].
(3) EVs can be modified to present any antigen of interest through the use of engineering techniques. Engineered EVs have been demonstrated to be safe, flexible, and an efficient strategy for a vaccine design [74].
(4) The strategy of presenting the myelin antigens inside the EVs is safer than the infusion of free peptides into the circulation. Although intravenous tolerance induction of free peptides has shown therapeutic effects in animal models of EAE, the safety of this approach remains a matter of concern as its repeated injections can induce anaphylactic shock and death in a portion of mice. In this sense, although the efficacy of antigen-presenting EVs is similar to the infusion of free antigens in the circulation, EVs have been shown to be safer. This characteristic makes antigen-presenting EVs a safer and more efficient strategy for inducing tolerance in EAE [88].

As discussed above, there are numerous advantages of EV-based vaccines over conventional ones; however, there are still some issues that need to be faced for clinical application:

(1) In particular, the phases of the selection and validation of the optimal combination of antigens and adjuvants are in the initial stages of research. The self-myelin antigen in MS remains unknown, with the possibility that these antigens differ among patients. The identification of this myelin antigen is an essential requirement for developing the antigen-specific therapy [88].
(2) EVs should not include other immunogenic antigens besides the desired ones. EVs released from macrophages and dendritic cells present on their surface the MHC Ι and ΙΙ molecules and B7 co-stimulatory molecules and the adhesion protein ICAM-1. These molecules can lead to an undesired reaction of the immune system [74]. To prevent this reaction, the selection of EVs derived from MSCs would be interesting, given that they present a very small amount of immunogenicity markers.
(3) The undesirable induction of further molecular responses in the EV recipient cells that could reduce the vaccine’s efficacy should also be assessed [74].

Despite these points, the findings obtained with EVs undoubtedly suggest the great potential of antigen-presenting EVs as a probable novel vaccine strategy. The generation of engineered EVs with a marked cell tropism towards the target immune system has allowed the development of nano-based antigen delivery system technologies, laying the foundations for a promising branch of personalised medicine.

## 7. Conclusions

In recent years, many studies have focused on EVs, and the promising results indicate a range of possibilities for their use as biomarkers, therapeutics, and, more recently, as vaccines. EVs have demonstrated an important role as biomarkers associated with disease activity and types of MS. The study of EVs isolated from various cell subpopulations from the immune and nervous system could provide information regarding the pathological processes of inflammation, astrocyte and microglia reaction, BBB permeability, leukocyte transendothelial migration, and untimely neuronal and synaptic damage. Current research also considers EVs to be a good biomarker of response to treatment. Thus, circulating EVs could provide information about response/failure to INF-β and fingolimod in patients with MS. Moreover, in natalizumab-treated patients, EVs could act as a marker of leukocyte transendothelial migration blockage. The content of EVs in INF-β and fingolimod-treated patients play a role in immune regulatory activity and in transendothelial migration of leukocytes to the CNS. Moreover, EVs have been considered not only as biomarkers, but also as potential treatments for MS. EVs from MSCs promote brain repair and immunomodulation in experimental animal models of MS. Last but not least, EVs could be a marker of risk of JCPyV infection after long periods of DMT administration. This assessment could improve the decision-making regarding treatment selection, initiation, change, and discontinuation. Finally, therapeutic approaches for restoring immune tolerance in CNS autoimmunity using “EV-based vaccines” for MS is under consideration in 2021.

## Figures and Tables

**Table 1:** Most commonly used methodologies to isolate EVs with their advantages, disadvantages and preferable applications.

Isolation Technique	Advantages	Disadvantages	Preferable Applications
Differential centrifugation	This technique is inexpensive, easy to implement and produces a high yield	Requires specialised equipment, produces excess contaminants and can damage EVs	Cell culture supernatants
DGU	DGU is inexpensive and produces high purity EVs	Requires specialised equipment and training and presents low throughput, low scalability and a low yield	Cell culture supernatants and biofluids (i.e., blood) if very high purity is not mandatory
SEC	SEC is scalable, induces good separation, preserves EV integrity, and removes soluble proteins and small molecules	SEC has limited sample capacity and requires sample dilution	Cell culture supernatants and biofluids (i.e., blood) if very high purity is not mandatory and where EV integrity is required
Bidimensional: DGU + SEC	These techniques produce very high purity	Requires specialised equipment and training and presents low throughput, low scalability, and a low yield	Biofluids, particularly interesting for blood
Precipitation	This technique is inexpensive, highly scalable, rapid and easy to implement	Precipitation produces low purity, isolates soluble non-EV material and the precipitation reagent needs to be removed	Cell culture supernatants, when low purity is acceptable (i.e., proven therapeutic effect)
Immunoisolation	This technique is rapid, produces high purity EVs and does not require specialised equipment	Immunoisolation is expensive, dependent on specificity of surface markers, presents low throughput and low yield, and affinity reagents need to be removed	Biofluids, particularly interesting for blood

*Abbreviations*: DGU, density gradient Ultracentrifugation; SEC, size exclusion chromatography.

**Table 3:** Advantages and disadvantages of using EV-based vaccines as antigen-presenting strategies for MS.

Advantages	Disadvantages
Antigen-presenting EVs target the immune system in an antigen-specific manner, leaving the rest of the immune system intact, thus reducing potential adverse effects.	The phases of the selection and validation of the optimal combination of antigens and adjuvants are in the initial stages of research.
EVs can be modified to present any antigen of interest thought the use of engineering techniques.	The self-myelin antigen in MS remains unknown, with the possibility that these antigens differ among patients.
EVs derived from MSCs present a low basal immunogenic profile. This characteristic guarantees the absence of immune system reactions when they are intravenously administered.	EVs should not include other immunogenic antigens such as MHC Ι and ΙΙ molecules and B7 co-stimulatory molecules and the adhesion protein ICAM-1 on their surface that can lead to an undesired reaction of the immune system.
Presenting the myelin antigens inside the EVs is safer than the infusion of free peptides into the circulation as the double lipid bilayer protects them from producing an immune system reaction.	The undesirable induction of further molecular responses in the EV recipient cells that could reduce the vaccine’s efficacy should also be assessed.

## Data Availability

Not applicable.
